# Farnesol-Loaded Nanoliposomes Inhibit Inflammatory Gene Expression in Primary Human Skeletal Myoblasts

**DOI:** 10.3390/biology11050701

**Published:** 2022-05-02

**Authors:** Eva Mückter, Maria Lozoya, Aline Müller, Volkmar Weissig, Mahtab Nourbakhsh

**Affiliations:** 1 Department of Geriatric Medicine, RWTH Aachen University Hospital, 52074 Aachen, Germany; emueckter@ukaachen.de (E.M.); almueller@ukaachen.de (A.M.); 2 College of Pharmacy, Midwestern University, Glendale, AZ 85308, USA; mlozoy@midwestern.edu (M.L.); vweiss@midwestern.edu (V.W.)

**Keywords:** farnesol, liposomes, nanovesicles, human, primary myoblasts, fatty acids, inflammation, cytokines, chemokines

## Abstract

**Simple Summary:**

Obesity leads to the accumulation of free fatty acids (FFAs) and inflammation in skeletal muscle, which results in the loss of muscle mass and function. Farnesol is a natural hydrophobic compound from plants with poor bioavailability. We used a liposomal delivery system to direct farnesol into muscle cells and examined its possible beneficial effects on restraining inflammation. Our data strongly suggest that farnesol acts as a potent inhibitor of inflammatory gene expression in human myoblasts.

**Abstract:**

There is a substantial unmet need for the treatment of skeletal muscle mass loss that is associated with aging and obesity-related increases in FFA. Unsaturated FFAs stimulate the inflammatory gene expression in human skeletal myoblasts (SkMs). Farnesol is a hydrophobic acyclic sesquiterpene alcohol with potential anti-inflammatory effects. Here, we created farnesol-loaded small unilamellar (SUVs) or multilamellar lipid-based vesicles (MLVs), and investigated their effects on inflammatory gene expression in primary human skeletal myoblasts. The attachment of SUVs or MLVs to SkMs was tracked using BODIPY, a fluorescent lipid dye. The data showed that farnesol-loaded SUVs reduced FFA-induced IL6 and LIF expression by 77% and 70% in SkMs, respectively. Farnesol-loaded MLVs were less potent in inhibiting FFA-induced IL6 and LIF expression. In all experiments, equal concentrations of free farnesol did not exert significant effects on SkMs. This report suggests that farnesol, if efficiently directed into myoblasts through liposomes, may curb FFA-induced inflammation in human skeletal muscle.

## 1. Introduction

The multifactorial disorder of sarcopenic obesity is characterized by the loss of skeletal muscle mass, and by obesity [1]. Clinical studies have suggested a direct correlation between the increased intramuscular fat tissue and degenerative decline of skeletal muscle mass and strength [2,3]. The high level of inflammatory cytokines produced by adipose tissue was suggested to accelerate muscle catabolism [4,5,6,7]. Animal studies have demonstrated that increased intramuscular lipid accumulation compromises muscle protein anabolism through cellular dysfunction [8,9]. Furthermore, free fatty acids (FFAs) and inflammatory mediators co-accumulate in the skeletal muscle of sarcopenic rats [10]. In organisms, FFAs are released from different types of cells upon lipolysis. FFAs differ by their chain length and the configuration of their unsaturated double bonds. FFAs were shown to play a pivotal role in signaling and gene activation in many different cell lines [11,12,13,14].

Skeletal muscle tissue contains quiescent myogenic cells, called satellite cells, which differentiate to skeletal myoblasts upon activation. Myoblasts can replicate and fuse to form myotubes and myofibers, and thereby play a key role in muscle tissue regeneration. Recently, we reported that unsaturated C16 and C18 FFAs induce the expression of inflammatory genes IL6, CXCL8, LIF, and CXCL12 in primary human skeletal myoblasts (SkMs) specifically [15].

Farnesol is an essential oil component from various plants with reported positive impacts on different inflammatory conditions like atherosclerosis and allergic asthma [16,17,18]. Farnesol, which is practically insoluble in water, was applied as a dietary supplement in previous human and animal studies. For more detailed experimental studies in cell culture, lower levels of farnesol were dissolved in methanol, or by adding solubilizing polysorbate 80 [18]. Farnesol dissolved in methanol led to the upregulation of inflammatory genes in human and murine cells in vitro [19,20]. However, farnesol solubilized using polysorbate has revealed significant anti-inflammatory effects in animal studies, as well as in macrophage cultures [16,17,21,22,23,24]. These conflicting results have raised concerns that methanol could be responsible for the upregulation of pro-inflammatory genes in vitro. Nevertheless, the chemical and physical characteristics of farnesol have limited further study of its safety, efficacy, and effectiveness as an anti-inflammatory compound.

Lipid-based vesicles, called liposomes, are useful tools for compound stabilization, improving cellular uptake, and directing compounds to specific target sites [25,26]. Liposomes consist of single or multiple concentric lipid bilayers that can be composed of cationic, anionic, or neutral lipids [26]. In the current study, we created farnesol-loaded liposomes and examined their inhibitory effects on the FFAs-induced expression of the inflammatory genes IL6, CXCL8, LIF, and CXCL12 in primary human skeletal myoblasts.

## 2. Materials and Methods

### 2.1. Fatty Acids (FAs)

As described previously, six mM analytical grade fatty acids C16[1]c and C18[2]c (Biotrend Chemikalien GmbH, Cologne, Germany) were conjugated to 2.4 mM FA-free bovine serum albumin (BSA, PAN Biotech, Aidenbach, Germany) at a 1:2.5 ratio in water by incubation at 50 °C for 5 min [27]. An equivalent BSA solution was prepared without FAs for control experiments.

### 2.2. Nanoliposome Preparation and Quantification

Liposomes (SUVs and MLVs) were prepared from L-α-phosphatidylcholine (PC) and 1,2-dioleoyl-3-trimethylammonium-propane (DOTAP) in 98:2% molar ratios (Avanti Polar Lipids Inc., Alabaster, AL, USA) and 4 mM farnesol (Sigma-Aldrich Inc., St. Louis, MI, USA) using the lipid film hydration method [28]. Briefly, all lipids and farnesol were solved in chloroform and mixed at desired ratios. A rotary vacuum evaporator (Yamato, RE-46, Santa Clara, CA, USA) was utilized to evaporate chloroform completely and to form a thin film of lipids, with or without farnesol. A total of 5 mM HEPES solution (4-(-2-hydroxyethyl) piperazine-1-ethane sulfonic acid) pH 7.4 was added to the films to obtain a final lipid concentration of 20 mg/mL, with or without 4mM farnesol, respectively. To obtain control-SUV and farnesol-SUV, the liposomal suspensions were sonicated (Sonic Dismembrator Model 100, Fisher Scientific, 5 W) for 45 min on ice and then centrifuged at 1550× *g* (RCF) for 15 min at 4 °C to remove possibly created titanium particles. Control-MLV and farnesol-MLV were created by the extrusion method. Briefly, the liposomal suspensions were extruded 13 times through a thin polycarbonate membrane filter (pore size 0.2 µm) using an extruder (micro extruder, Avanti Polar Lipids Inc.) to homogenize the size of the MLVs. The hydrodynamic diameters of the liposomal nanoparticles were measured by dynamic light scattering (DSL) in triplicate using a Nano ZS Zetasizer (Malvern Panalytical, Westborough, MA, USA) at 25 °C. In this study, the size reported is based on the average particle diameter by volume. Each measurement was performed on freshly prepared samples without dilution. SUVs and MLVs were sterile filtered using 0.22 and 0.45 µm pore size before being applied to cells.

### 2.3. Reverse-Phase High-Performance Liquid Chromatography (HPLC)

An Agilent Infinity 1200 series HPLC system (Agilent Technologies, Santa Clara, CA, USA), comprising a binary pump, an autosampler, a degasser, a column compartment, and a DAD detector, was employed to separate and detect the farnesol. A Waters Novapak C18 column 4 µm column (3.9 mm inner diameter and 150 mm length; Waters Milford, MA, USA) was used as the stationary phase. The chromatographic analyses were implemented in isocratic mode using a methanol and water mixture (80:20, *v*/*v*) for the mobile phase at a flow rate of 0.6 mL/min and UV acquisition (210 nm). The sample injection volume was 5 µL of each 4 mM free farnesol reference, farnesol-SUV, or farnesol-MLV suspension containing 4 mM farnesol. The analyses were run for 10 min at 25 °C. The farnesol retention time was 4.28 (±0.2) min. Samples were analyzed without any treatment. After three independent experiments, the amounts of farnesol recovered from vesicles were compared to that from the reference solution, which was set to 100%. The liposomal encapsulation efficiency (LEE%) was calculated using following formula: LEE% = ((the amount of farnesol in the nanovesicle suspension/the total quantity of drug added initially during preparation) × 100).

### 2.4. Cell Culture

Primary skeletal muscle myoblasts (SkMs) derived from a nineteen-year-old healthy male human donor were obtained from Lonza, Basel, Switzerland and maintained in SkGM2 BulletKit Medium (Lonza) at 37 °C and 5% CO_2_, as described previously [15]. SkMs (5000 cells/cm^2^) were seeded on 6-well (multiplex protein quantification) or 96-well plates (multiplex mRNA quantification) and maintained for 48 h, and then incubated in SkGM2 BulletKit Medium with 20 µM farnesol or a 1:200 dilution of SUVs or MLVs loaded with or without farnesol. After 24 h, cells were washed with HEPES BSS (Lonza: CC-5024) and then incubated with 50 µM FFA in BSA or BSA (control) in SkGM2 BulletKit Medium. Cells were then harvested after 24 h for mRNA quantification or after 48 h for protein quantification, as described previously [15].

### 2.5. Monitoring Mitochondrial Abundance and Function

SkMs were seeded on 24-well plates (2500 cells/well) and treated with free farnesol, farnesol-SUV, farnesol-MLV, control-SUV, or control-MLV, or left untreated. After 24 h, cells were incubated with 1 µM MitoTracker Orange CM-H2TMRos (Thermo Fisher Scientific, Waltham, MA, USA) or 200 nM MitoTracker Red CMXRos (Thermo Fisher Scientific) in DMEM (P04-0359, PAN Biotech, Aidenbach, Germany) for 60 or 30 min, respectively. An automated inverted microscope (DM4000B, Leica Microsystems) with 515–560-nm filter was used to capture the images.

### 2.6. Lipid Vesicle Imaging

SkMs were seeded on 24-well plates (2500 cells/well) and left untreated or treated with free farnesol, farnesol-SUV, farnesol-MLV, control-SUV, or control-MLV. After 24 h, cells were carefully washed in HEPES BSS (Lonza) and incubated with 2 µM BODIPY 493/503 (Thermo Fisher Scientific) in serum-free DMEM (P04-03590, PAN Biotech) for 30 min at 37 °C. Cells were washed in HEPES BSS (Lonza) before imaging. An automated inverted microscope (DM4000B, Leica Microsystems) with 450–490-nm filter was used to capture the images.

### 2.7. Multiplex Protein Quantification

SkMs were seeded on 6-well plates (48,000 cells/well) and left untreated or were treated with free farnesol, farnesol-SUV, farnesol-MLV, control-SUV, or control-MLV. After 24 h, cells were washed with HEPES BSS (Lonza: CC-5024) and left unstimulated or stimulated with fatty acids C16[1]c or C18[2]c. After 48 h, cells were washed with HEPES BSS (Lonza: CC-5024) and lysed in 400 µL Procarta Plex Cell Lysis Buffer (Thermo Fisher Scientific). The protein concentrations of SkM lysates were determined using Pierce 660 nm Protein Assay Kit (22662, Thermo Fisher Scientific), according to the manufacturer’s instructions. Customized ProcartaPlex immunoassays (Thermo Fisher Scientific) were performed to quantify the concentration of different markers in 25 µL of cell lysates using Luminex xMAP technology-based Magpix (Thermo Fisher Scientific), according to the manufacturer’s instructions. The amount of each marker was normalized to one µg of total cellular proteins.

### 2.8. Multiplex mRNA Quantification

SKMs were seeded on 96-well plates (1600 cells/well) and left untreated or were treated with free farnesol, farnesol-SUV, farnesol-MLV, control-SUV, or control-MLV. After 24 h, cells were washed with HEPES BSS (Lonza: CC-5024) and left unstimulated or were stimulated with fatty acids C16[1]c or C18[2]c. After 24 h, cells were lysed in 150 µL Quantigene buffer. Quantigene Plex assays (Thermo Fisher Scientific) were used to quantify IL6, CXCL8, LIF, and CXCL12 mRNAs expression in 33 µL of SkM lysates by direct hybridization to specifically designed capture extender (CE), label extender (LE), and blocking probes (BL). The sequences are provided in the Supplementary Materials, Figure S1. For quantitative comparison of mRNAs among the samples, the level of GAPDH and GUSB mRNAs were determined and compared as internal controls. Sample preparation and analysis were performed using Magpix, according to the manufacturer’s instructions (Thermo Fisher Scientific).

### 2.9. Statistical Analysis

The relative means of values were normalized to the untreated control and presented ±  standard deviations. ANOVA multiple comparison testing with Bonferroni’s multiple comparisons were employed to compare the results from parallel experiments. A *p*-value of ≤0.05 was considered as statistically significant (*).

## 3. Results

### 3.1. Farnesol Can Be Efficiently Encapsulated in PC[soy]/DOTAP-Based SUV and MLV

**Figure 1:**
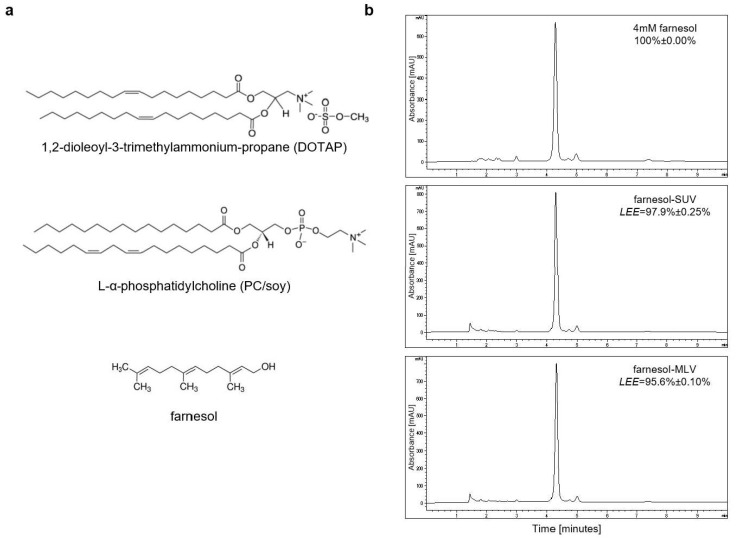
Preparation of control and farnesol vesicles. (**a**) The molecular structure of DOTAP, PC/soy, and farnesol are presented; (**b**) Chromatograms represent one of three independent HPLC analyses of free farnesol (4 mM, upper panel), farnesol-SUV (4 mM, middle panel), and farnesol-MLV (4 mM, lower panel) are presented. Mean liposomal encapsulation efficiencies (LEE) ± SD were calculated from three independent runs and indicated at the top-right of each chromatograph. High resolution images were provided in Supplementary Materials, Figure S2.

Farnesol is a sesquiterpene alcohol with a molecular weight of 222.372 g/mol. In this study, we used the commercially available trans, trans-farnesol, which is one of four isomers found in nature (Figure 1a). Farnesol is highly hydrophobic and practically insoluble in water in the absence of organic solvents or solubilizing detergents. To study the biological effects of farnesol in adherent primary cell culture, we established a liposome-based delivery system using 1,2-dioleoyl-3-trimethylammonium-propane (DOTAP) and L-α-phosphatidylcholine (PC/soy) (Figure 1a). DOTAP was successfully utilized to form vesicles for the delivery of unstable siRNAs into cancer cells [29]. We established two structurally different vesicles, SUVs and MLVs, comprising farnesol (farnesol-SUVs and farnesol-MLVs), or left blank as control vesicles (SUVs and MLVs). The formation of the vesicles was determined using dynamic light scattering (DSL). The analysis of the vesicles’ size distribution by volume revealed no significant difference between control-SUV and farnesol-SUV and a minor difference between control-MLV and farnesol-MLV (Table 1 and Supplementary Figure S3).

Next, we compared the concentration of farnesol in liposomes with the initial 4 mM farnesol reference solution using reverse-phase high-performance liquid chromatography (HPLC), as reported previously with some modifications [30,31]. Farnesol signals were detected with the mobile phase consisting of 80% methanol, which dissolves lipid bilayers. We observed nearly identical chromatograms of farnesol-SUV, farnesol-MLV, and the initial farnesol solution (Figure 1b). Thus, the prepared farnesol-MLV and farnesol-SUV emulsions harbor nearly 3.824 and 3.916 mM farnesol, respectively. The calculated encapsulation efficiencies for farnesol-MLV and farnesol-SUV were 95.6 ± 0.10% and 97.9 ± 0.25%, respectively.

### 3.2. DOTAP-PC/Soy Vesicles Do Not Affect Mitochondrial Abundance or Oxidative Activity in SkMs

**Figure 2:**
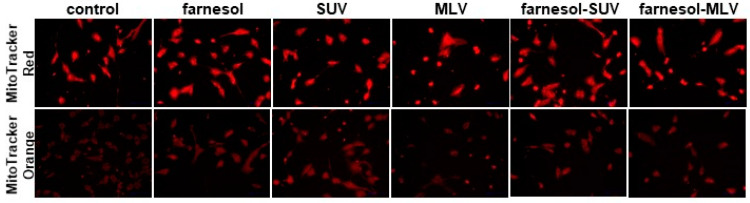
Abundance and oxidative activity of mitochondria in human primary myoblasts. SkMs were left untreated (control) or treated with farnesol, SUV, MLV, farnesol-SUV, or farnesol-MLV, as indicated. After 24 h, SkMs mitochondria were stained using MitoTracker Red (upper panel) or Orange (lower panel). The presented images were captured at the same settings and exposure times and are representative of three independent sets of experiments. High resolution images are provided in the Supplementary Materials, Figure S4.

Recently, we established a new experimental approach using primary human skeletal myoblasts (SkMs) to address the potency of free fatty acids (FFAs) in the regulation of inflammatory genes [15]. Here, we used this approach to compare the effects of different liposomes on the inflammatory response of SKMs. Elevated lipid levels have previously been associated with loss of mitochondrial function and integrity in muscle cells [32]. Therefore, we used two different derivatives of rosamine-based MitoTracker dyes to examine a possible negative effect of liposomes on the mitochondrial function in SkMs. MitoTracker Red is a fluorescent dye that accumulates in mitochondria, depending on membrane potential. MitoTracker Orange is a nonfluorescent reduced derivate that accumulates in mitochondria but fluoresces upon oxidation. In all experiments, we did not observe a significant effect of control-MLV, control-SUV, farnesol-SUV, farnesol-MLV, or free farnesol on the abundance or oxidative function of mitochondria compared to untreated cells (Figure 2). Thus, the generated liposomes and their lipid components do not affect the mitochondrial abundance or oxidative activity in SkMs under the established experimental conditions.

### 3.3. DOTAP-PC/Soy Vesicles Equally Attach to SkMs

**Figure 3:**
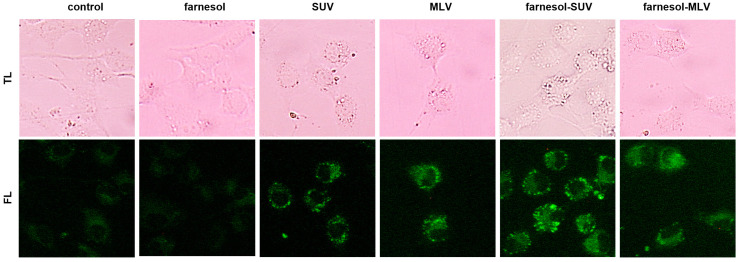
Nanovesicles equally attach to SkMs. SkMs were not treated (control) or treated with farnesol, SUV, MLV, farnesol-SUV, or farnesol-MLV, as indicated. After 24 h, SkMs were stained using BODIPY. Transmitted light (TL, **upper** panel) and fluorescence (FL, **lower** panel) images were captured at the same settings of magnification and exposure times. High resolution images were provided in Supplementary Figure S5.

To track the attachment of liposomes to living cells, we used a green hydrophobic fluorescent dye that can stain lipids and other lipophilic compounds. SkMs were treated with different vesicles or left untreated before staining. As shown in Figure 3, free farnesol and nontreated cells showed marginal fluorescent signals within SkMs cytoplasm. However, all vesicles revealed a substantial accumulation of green fluorescent signals at the SkMs cell membrane. Notably, SUVs exhibited more protuberant fluorescent signals than MLVs, which may be due to the structural difference between SUVs and MLVs (Figure 3).

### 3.4. Unsaturated FFAs Modulate the Expression of Inflammatory Proteins in SkMs

Among 39 different inflammatory proteins, we identified IL6, CXCL8, LIF, and CXCL12 to be differentially expressed in the primary human skeletal myoblasts (SkMs) in response to specific species of free fatty acids (FFAs) [15]. We chose primary cells because their characteristics and activities are highly related to that of the source tissue. Due to their limited lifespan, we used a number of identical stocks of SkMs from a single healthy adult to perform three independent sets of experiments.

**Figure 4:**
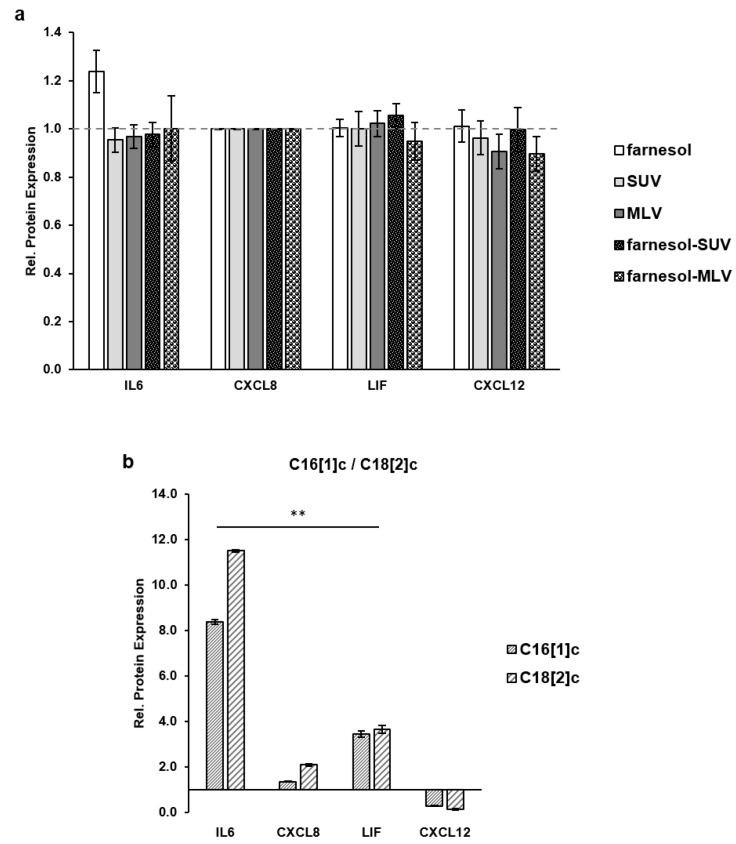
FFAs, but not nanovesicles, affect the inflammatory protein expression. (**a**) SkMs were left untreated or treated with farnesol, SUV, MLV, farnesol-SUV, or farnesol-MLV for 24 h; (**b**) SkMs were treated with FFA C16[1]c or C18[2]c or left untreated for 48 h. SkM cell extracts were subjected thrice to multiplex protein quantification. The relative protein expression was obtained by comparing the level of each marker in treated cells to its expression level in untreated cells (no farnesol or liposomes in **a** and no fatty acids in **b**), which was set to 1 (dashed gray lines in **a**,**b**, respectively). The mean values ± SD from three independent experiments are presented (Supplementary Figure S6). Statistical significance was calculated using one-way ANOVA with Bonferroni’s multiple comparison test. *p* ≤ 0.01 (**).

Equal amounts of SkM extracts were subjected to a multiplex quantification panel for IL6, CXCL8, LIF, and CXCL12. In an initial set of control experiments, we eliminated a possible effect of farnesol, control (SUV and MLV), or farnesol vesicles (farnesol-SUV and farnesol-MLV) on IL6, CXCL8, LIF, and CXCL12 expression in the absence of FFAs (Figure 4a). Therefore, the level of each protein marker in the cells, which were treated with farnesol, MLV, SUV, farnesol-SUV, or farnesol-MLV, was normalized to its expression level in untreated control cells to obtain the relative protein expression (Figure 4a). The data from three independent experiments show that none of these treatments affect the expressions of IL6, CXCL8, LIF, and CXCL12. Next, we stimulated SkMs with two different FFAs, C16[1]c and C18[2]c. This abbreviated nomenclature depicts the number of carbon atoms in the FA chain followed by the number of unsaturated carbon double bonds in square brackets and the cis configuration indicated by the letter “c”. To obtain the relative activation of protein expression by FAs, the level of each marker in C16[1]c or C18[2]c treated cells was normalized to its expression level in the untreated control cells (Figure 4b). The data show that both FAs induce the expression of IL6, CXCL8, and LIF, but inhibit CXCL12 expression in SkMs (Figure 4b).

### 3.5. Farnesol Inhibits the Expression of Inflammatory Proteins in Response to FFAs

**Figure 5:**
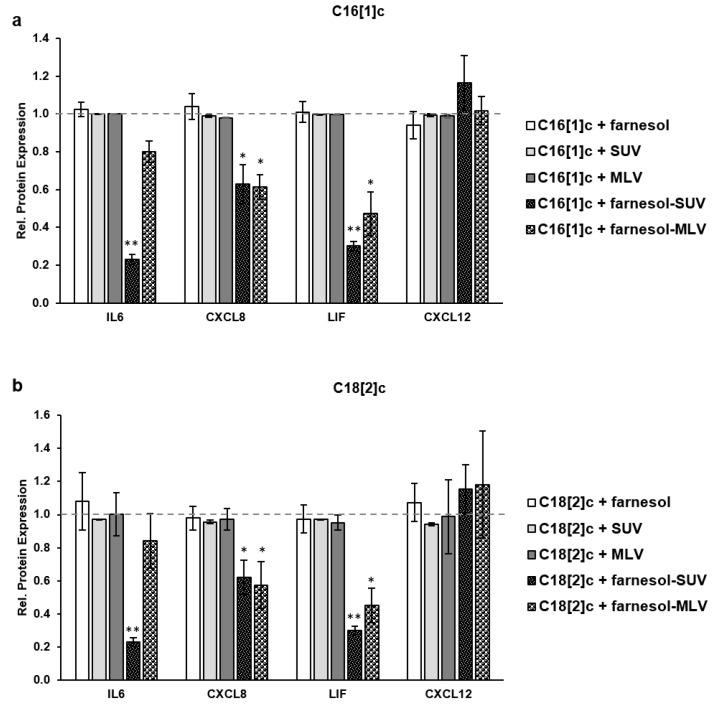
Farnesol inhibits the FFA-induced expression of inflammatory proteins. SkMs were left untreated or treated with farnesol, SUV, MLV, farnesol-SUV, or farnesol-MLV for 24 h; (**b**) SkMs were washed and treated with C16[1]c (**a**) or C18[2]c (**b**) or left untreated for 48 h. SkM extracts were then analyzed using multiplex cytokine quantification assays thrice. The relative protein expression was obtained by comparing the level of each marker in farnesol-, or liposome-treated cells to its expression level in FFA-stimulated cells (C16[1]c in **a** and C18[2]c in **b**), which was set to 1 (dashed gray lines, **a**,**b**). The mean values ± SD from three independent experiments are presented (Supplementary Materials, Figure S6). Statistical significance was calculated using one-way ANOVA with Bonferroni’s multiple comparison test. *p* ≤ 0.05 (*). *p* ≤ 0.01 (**).

The BODIPY experiments suggested that the liposomes accumulated at the SkMs membrane about 24 h after they were added to the SkMs supernatants. However, FA signaling in SkMs was faster, involving multiple steps of signal transduction, gene activation, transcription, and protein translation within 48 h. To achieve a sufficient uptake of farnesol before gene activation, SkMs were treated with free farnesol or liposomes first and then stimulated with C16[1]c and C18[2]c FAs after 24 h. To obtain the relative protein expression in this set of experiments, the level of each protein marker in SkMs, which were treated with free farnesol or liposomes and then stimulated with FAs, was normalized to its expression level in SkMs, which were treated with C16[1]c (Figure 5a), or C18[2]c (Figure 5b), exclusively. The data showed that C16[1]c- or C18[2]c-stimulated IL6 and LIF expression was most significantly inhibited by farnesol-SUV (Figure 5a,b). The effects of farnesol-MLV were less prominent than those of farnesol-SUV. As expected, we did not observe any effects on CXCL12 expression. Furthermore, farnesol-SUV and farnesol-MLV revealed a minor effect on CXCL8 expression, which was less responsive to FFAs (Figure 5b). It is important to note that free farnesol and control-SUV and control-MLV did not affect the expression of IL6, CXCL8, LIF, or CXCL12 (Figure 5a,b).

### 3.6. Farnesol Inhibits the Transcription of IL6, CXCL8 and LIF Genes

**Figure 6:**
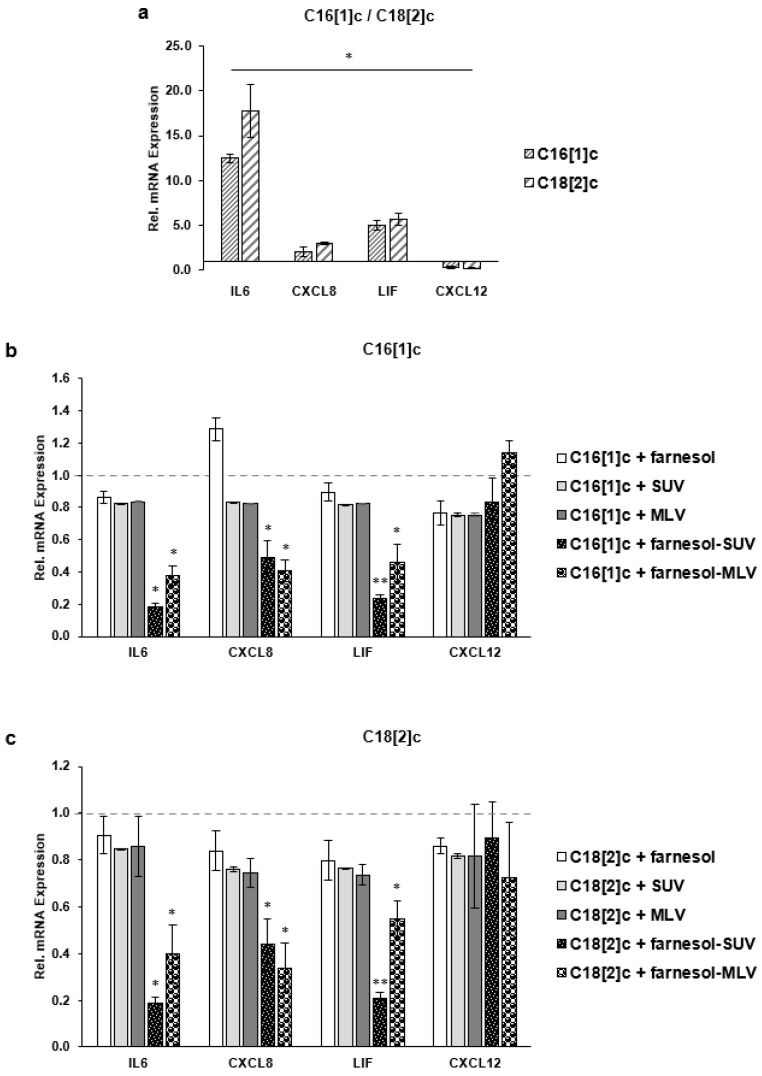
Farnesol inhibits the FFA-induced transcription of inflammatory genes. SkMs were left untreated or treated with farnesol, SUV, MLV, farnesol-SUV, or farnesol-MLV for 24 h; (**b**) SkMs were washed and treated with C16[1]c (**a**,**b**), C18[2]c (**a**,**c**) or left untreated for 24 h in three independent sets of experiments. SkM extracts were analyzed in triplicates using multiplex mRNA quantification to determine the expression level of IL6, CXCL8, LIF, and CXCL12 mRNAs normalized to the level of GAPDH and GUSB mRNAs. To obtain the relative FA-mediated activation of expression, the expression of each marker mRNA in FA-treated cells was compared to its expression level in untreated cells (**a**). To obtain the relative inhibition by farnesol, the expression of each marker mRNA in farnesol- or liposomes-treated cells was compared to its expression in C16[1]c-treated (**b**) or C18[2]c-treated cells (**c**).Thus, the expression level in untreated control cells (**a**), C16[1]c-treated cells (**b**), or C18[2]c-treated cells (**c**) was set to 1 (dashed gray line). The results are presented as the mean ± SD of the relative expression of designated markers in three independent experiments (white bars). Statistical significance was calculated using one-way ANOVA with Bonferroni’s multiple comparison test. *p* ≤ 0.05 (*). *p* ≤ 0.01 (**).

Next, we determined the expression levels of IL6, CXCL8, LIF, and CXCL12 mRNAs using a multiplex hybridization assay, which avoided possible variations due to reverse transcription of amplification. SkMs were treated as described above in three independent experiments (Section 3.4). We determined the abundance of each encoding mRNA by direct comparison with the mRNA level of two housekeeping genes. For a direct comparison of effects, the level of each mRNA in C16[1]c or C18[2]c treated cells was normalized to its expression level in the control cells, which were not treated with FA (Figure 6a). As shown in Figure 6a, C16[1]c and C18[2]c induced IL6, CXCL8, LIF, and CXCL12 gene expression at the transcriptional level. Next, SkMs were treated with free farnesol or liposomes first and then stimulated with C16[1]c and C18[2]c FAs after 24 h, as described above (Section 3.5). To obtain the relative protein expression in this set of experiments, the level of each mRNA in SkMs, which were treated with free farnesol or liposomes and then stimulated with FAs, was normalized to its expression level in SkMs, which were treated with C16[1]c (Figure 6b) or C18[2]c (Figure 6c), exclusively. Thus, the expression level of each mRNA in C16[1]c (Figure 6b) or C18[2]c cells (Figure 6c) was set to 1 (dashed gray lines). The data confirmed that C16[1]c- or C18[2]c-mediated IL6 and LIF expression was most significantly inhibited by farnesol-SUV (Figure 6b,c). Most importantly, farnesol mediated effects of IL6, CXCL8, and LIF mRNAs corresponded to its effects on protein expression described in Figure 5a,b. Together, our results suggested that farnesol modulates inflammatory gene transcription specifically in response to C16[1]c and C18[2]c, through the inhibition of signal transduction pathways.

## 4. Discussion

Some previous reports on the anti-inflammatory potential of farnesol were encouraging. However, its high hydrophobicity and volatility are critical challenges for more in-depth studies. Loading farnesol into liposomes is a promising alternative for its further characterization in different cellular or pathological contexts. Our study provides a useful strategy for the stabilization and delivery of farnesol into cells using liposomes. Another advantage of these vesicles is that they are easily traceable upon attachment to living cells. In addition, the data substantiate the anti-inflammatory impact of farnesol on primary human skeletal myoblasts. Farnesol inhibits the FFA-mediated induction of inflammatory genes, which is an important pathological hallmark of sarcopenic obesity [2].

In terms of liposome production, egg yolk and soybean are the most important sources of PC. We used PC from soy because egg yolk PC may contain traces of long-chain polyunsaturated fatty acids and sphingomyelin that may potentially activate inflammatory genes in skeletal myoblasts [15]. Indeed, soy PC-based vesicles were useful for the study of inflammatory gene responses, showing no unintended effects on IL6, CXCL8, LIF, or CXCL12 expression (Figure 5a). In general, SUVs and MLVs differ by the volume and number of bilayer membranes that determine their stability and the loaded amount of a compound [26,33]. According to the particle analysis, the inclusion of farnesol did not significantly affect the formation or size distribution of SUVs but led to a minor division of smaller-sized MLVs (Table 1).

Recent clinical studies revealed that obesity-induced excessive amounts of ectopic lipids trigger mitochondrial fission and quality in skeletal muscle fibers [34]. Given that the application of vesicles did not alter the mitochondrial abundance or activity in this study, we assume that the applied concentration of lipid components may be negligible compared to the reported accumulation of lipids in obese individuals over time. Our results disclose the effects of liposomes in SkMs within 24 h, which correspond to the 6th doubling cycle after isolation from tissue. The proliferation and viability of SkMs decline rapidly at the 10th doubling cycle. Thus, the short lifespan of SkMs does not allow the monitoring of liposome effects over an extended timeline.

For the first time, we used a useful tracer of lipid trafficking, BODIPY, to monitor and compare the attachment of vesicles to SkMs. This dye was previously used to detect cytoplasmic lipid droplets in myotubes after the fixation of myotubes with paraformaldehyde [35]. In a first attempt, the fixation of SkMs revealed no significant accumulation of fluorescent signals by liposomes (data not shown). Thus, we suggest that the attachment of liposomes to the cell membrane is sensitive to denaturing conditions. Under native conditions, the fluorescent signals were strikingly visible close to the cells after 24 h (Figure 3). Although the captured fluorescent signals indicate the location of accumulated lipids, they cannot specify the structural condition of the liposomes at this stage. We note that the accumulation of fluorescent signals on cells was slightly detectable two hours after the addition of liposomes into the cell culture medium (data not shown). Thus, BODIPY may be a resourceful and convenient dye for tracking the attachment of lipid-based vesicles to cells.

C16[1]c and C18[2]c FFAs activate IL6, CXCL8, and LIF genes to different extents. Although farnesol-SUV and farnesol-MLV inhibited these genes, a marked difference was noted between the magnitude of their actions on IL6 protein expression specifically. However, farnesol-SUV and farnesol-MLV significantly inhibited IL6 mRNA expression. Moreover, our results strongly suggest that farnesol primarily acts at the transcriptional level, as confirmed by a significant decrease in IL6, CXCL8, and LIF transcripts. However, we cannot exclude the possible additional effect of farnesol on the translation or stability of IL6 protein. It is also conceivable that the disparate effects on IL6 protein expression may partially result from the differential kinetics of farnesol release by MLVs and SUVs or the differential response of IL6 expression to a specific level of farnesol in cells. The data from HPLC analysis confirmed that MLVs and SUVs harbored nearly equal amounts of farnesol. However, the analysis of farnesol release rates into cells is highly complex and requires further examination.

Since the identification of farnesol, several studies reported on both pro- and anti-inflammatory effects of farnesol in vitro and in vivo, respectively. Although some of the reported inconsistencies can be attributed to the complexity of the inflammation process and the involvement of many different cell types and factors, the dosage and administration methods of farnesol play an important role. The use of SUV-farnesol may improve the application and cellular uptake of farnesol under experimental conditions and allow for the further investigation of its molecular actions in vitro and in vivo.

## 5. Conclusions

Excessive fat deposits increase the level of FFAs in skeletal muscle tissue, which leads to local inflammation and loss of skeletal muscle mass. This study provides a rationally designed delivery system for water-insoluble farnesol, which confers anti-inflammatory effects by inhibiting the activity of the relevant interleukin genes IL6, CXCL8, and LIF in human primary skeletal myoblasts. The findings of this study provide important insights into the possible therapeutic effects of farnesol against sarcopenic obesity and support its clinical development in future preclinical studies.

## Figures and Tables

**Table 1:** Physicochemical characterization of liposomes evaluated by DLS.

Liposomes	Composition	Size by Volume ± SD (nm)	Polydispersity Index
SUV	PCsoy/TAP (98:2)	27.3 ± 5.7	0.384
farnesol-SUV	PCsoy/TAP:4.0 mM Farnesol	33.77 ± 0.76	0.396
MLV	PCsoy/TAP (98:2)	322.6 ± 28.48	0.247
farnesol-MLV	PCsoy/TAP/4.0 mM Farnesol	195.7 ± 1.58	0.163

## Data Availability

The data presented in this study are available within the article and supplementary materials.

## Supplementary Materials

The following are available online at https://www.mdpi.com/article/10.3390/biology11050701/s1. Figure S1: Gene sequences used for Quantigene experiments; Figure S2: High resolution image of Figure 1b; Figure S3: Graphic presentation of the results from dynamic light scattering (DSL); Figure S4: Row data to Figure 4 and Figure 5; Figure S5: High resolution images of Figure 2; Figure S6: High resolution images of Figure 3.
