# Supplementary figures and images for: Farnesol-Loaded Nanoliposomes Inhibit Inflammatory Gene Expression in Primary Human Skeletal Myoblasts
Source: Biology (Basel). 2022 May 2;11(5):701. doi: 10.3390/biology11050701 (PMC9138524; doi:10.3390/biology11050701)

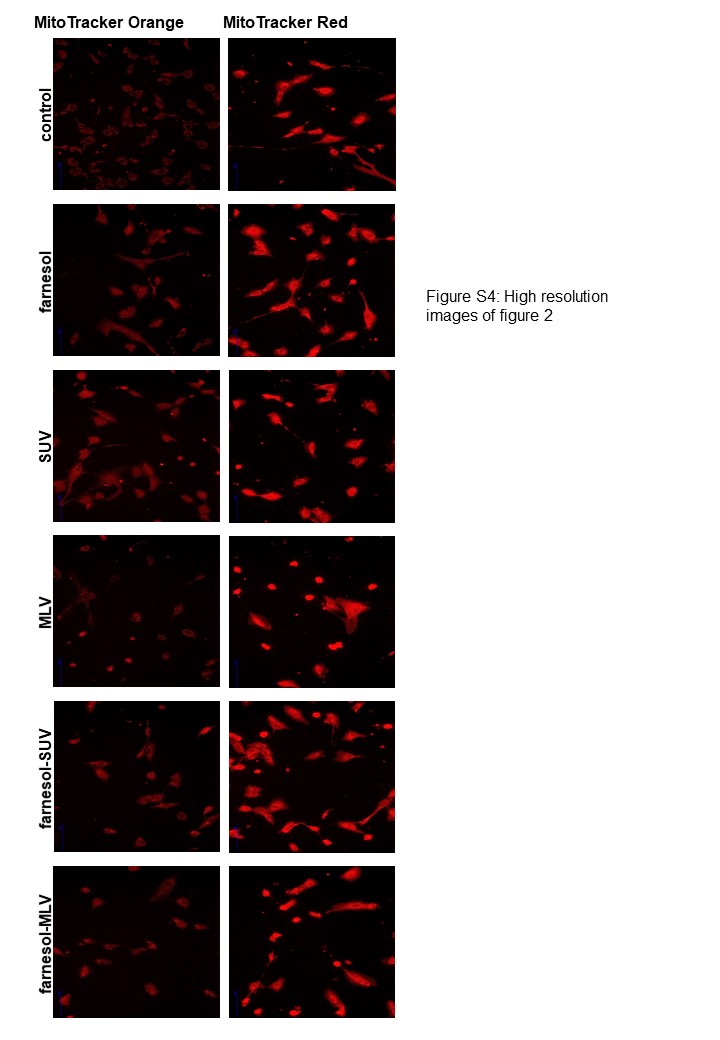

Supplement: Supplementary file 1 [file biology-11-00701-s001.zip › Folie10.JPG]

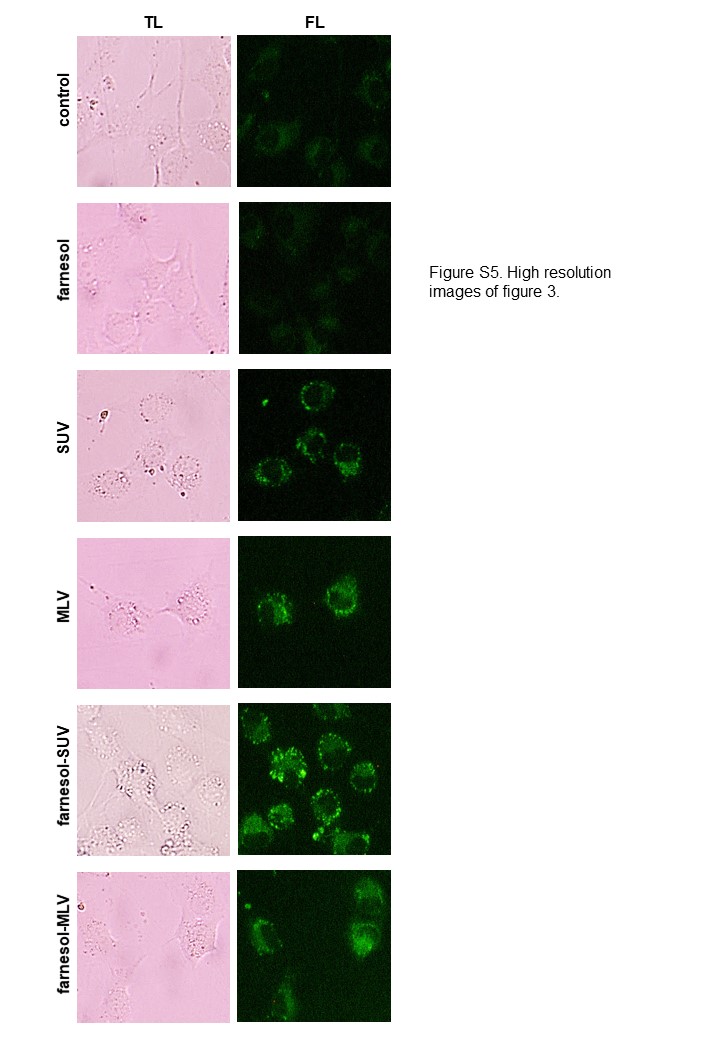

Supplement: Supplementary file 1 [file biology-11-00701-s001.zip › Folie11.JPG]

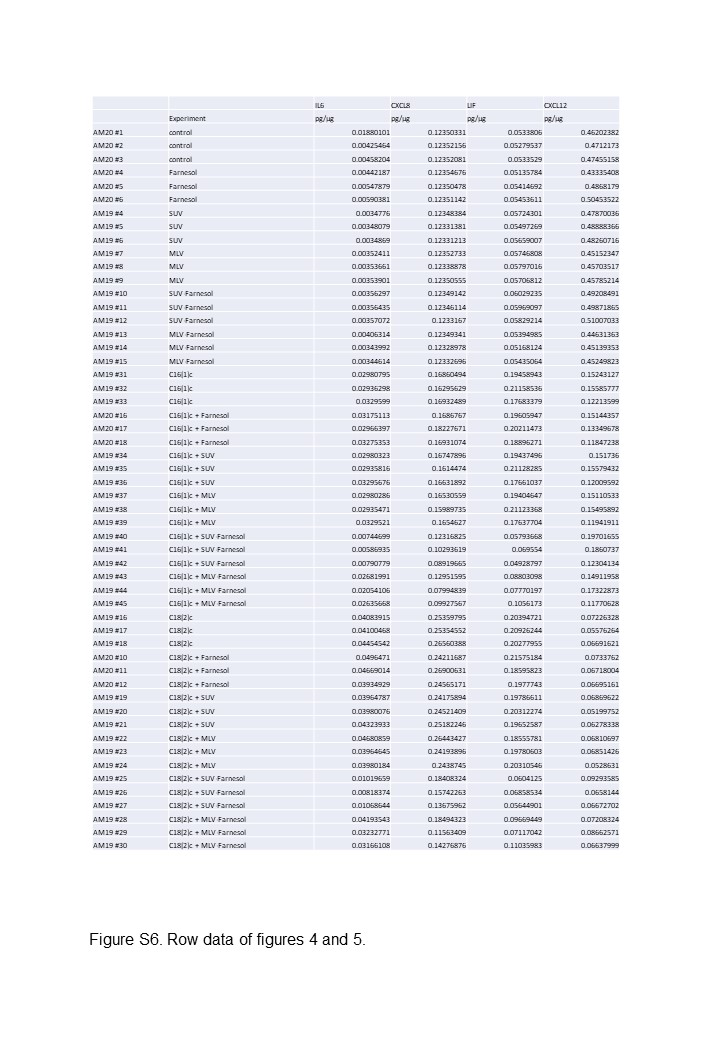

Supplement: Supplementary file 1 [file biology-11-00701-s001.zip › Folie12.JPG]

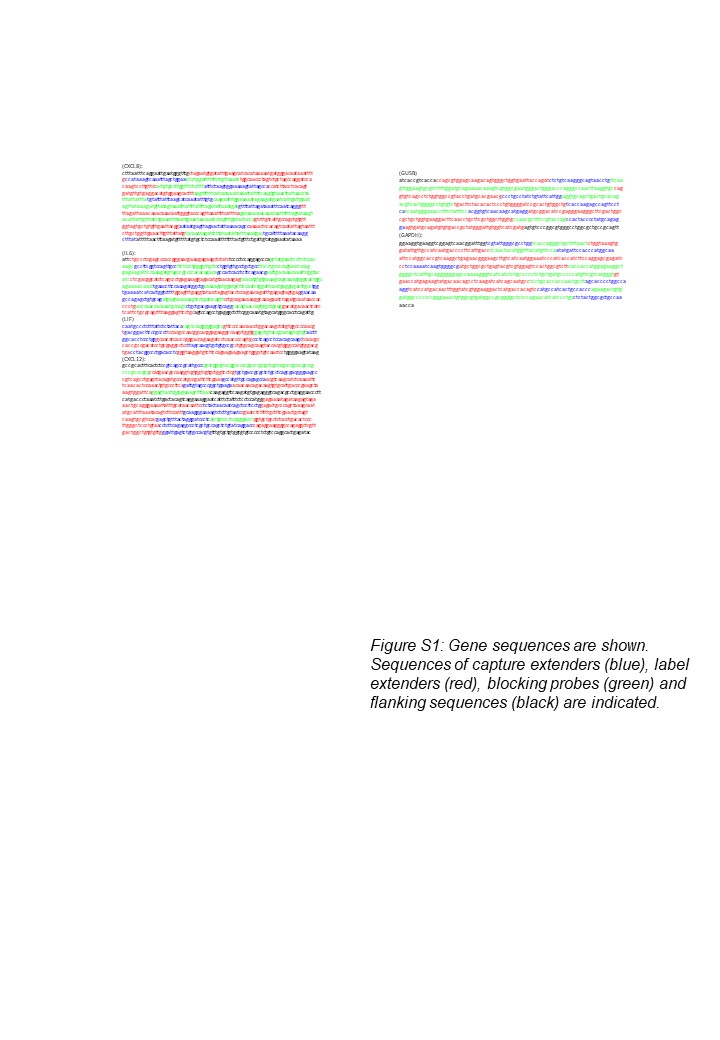

Supplement: Supplementary file 1 [file biology-11-00701-s001.zip › Folie7.JPG]

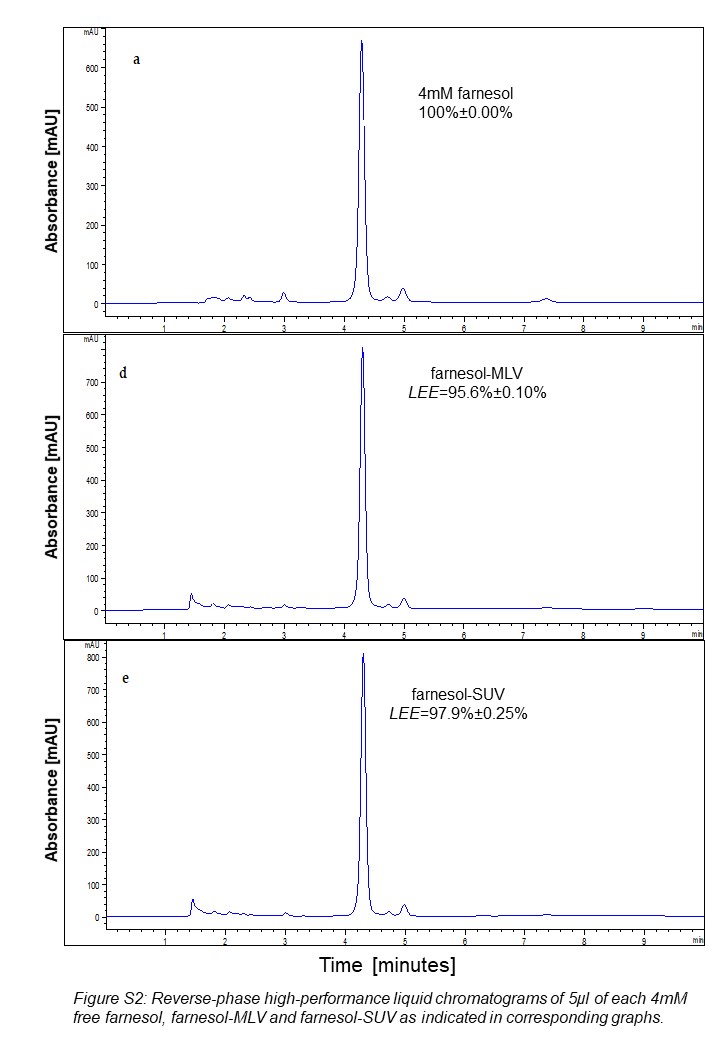

Supplement: Supplementary file 1 [file biology-11-00701-s001.zip › Folie8.JPG]

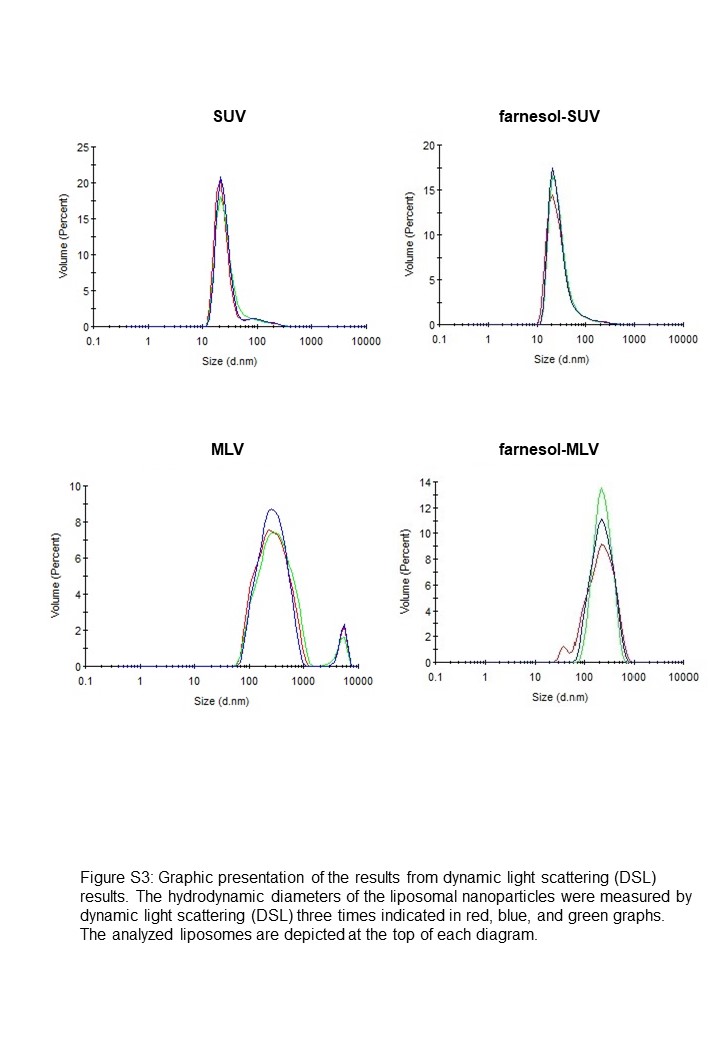

Supplement: Supplementary file 1 [file biology-11-00701-s001.zip › Folie9.JPG]
